# Opioid-free versus opioid-based anesthesia in pancreatic surgery

**DOI:** 10.1186/s12871-021-01551-y

**Published:** 2022-01-04

**Authors:** Stéphane Hublet, Marianne Galland, Julie Navez, Patrizia Loi, Jean Closset, Patrice Forget, Pierre Lafère

**Affiliations:** 1 Department of Anesthesiology, Université Libre de Bruxelles, CUB Érasme, Brussels, Belgium; 2 Department of Abdominal Surgery and Transplantation, Université Libre de Bruxelles, CUB Érasme, Brussels, Belgium; 3 Clinical Chair in Anaesthesia, University of Aberdeen, Aberdeen, UK

**Keywords:** Analgesics, Non-Narcotic / therapeutic use, Analgesics, Opioid / therapeutic use, Balanced Anesthesia / methods, Pain, Postoperative / drug therapy, Treatment Outcome

## Abstract

**Background:**

Opioid-free anesthesia (OFA) is associated with significantly reduced cumulative postoperative morphine consumption in comparison with opioid-based anesthesia (OBA). Whether OFA is feasible and may improve outcomes in pancreatic surgery remains unclear.

**Methods:**

Perioperative data from 77 consecutive patients who underwent pancreatic resection were included and retrospectively reviewed. Patients received either an OBA with intraoperative remifentanil (*n* = 42) or an OFA (*n* = 35). OFA included a combination of continuous infusions of dexmedetomidine, lidocaine, and esketamine. In OBA, patients also received a single bolus of intrathecal morphine. All patients received intraoperative propofol, sevoflurane, dexamethasone, diclofenac, neuromuscular blockade. Postoperative pain management was achieved by continuous wound infiltration and patient-controlled morphine. The primary outcome was postoperative pain (Numerical Rating Scale, NRS). Opioid consumption within 48 h after extubation, length of stay, adverse events within 90 days, and 30-day mortality were included as secondary outcomes. Episodes of bradycardia and hypotension requiring rescue medication were considered as safety outcomes.

**Results:**

Compared to OBA, NRS (3 [2–4] *vs* 0 [0–2], *P* < 0.001) and opioid consumption (36 [24–52] *vs* 10 [2–24], *P* = 0.005) were both less in the OFA group. Length of stay was shorter by 4 days with OFA (14 [7–46] *vs* 10 [6–16], *P* < 0.001). OFA (*P* = 0.03), with postoperative pancreatic fistula (*P* = 0.0002) and delayed gastric emptying (*P* < 0.0001) were identified as only independent factors for length of stay. The comprehensive complication index (CCI) was the lowest with OFA (24.9 ± 25.5 *vs* 14.1 ± 23.4, *P* = 0.03). There were no differences in demographics, operative time, blood loss, bradycardia, vasopressors administration or time to extubation among groups.

**Conclusions:**

In this series, OFA during pancreatic resection is feasible and independently associated with a better outcome, in particular pain outcomes. The lower rate of postoperative complications may justify future randomized trials to test the hypothesis that OFA may improve outcomes and shorten length of stay.

## Introduction

Pancreas cancer is currently the seventh leading cause of cancer death worldwide. A major concern is that the incidence of pancreatic cancer is increasing in the Western world. It is anticipated to become the second leading cause of cancer-related mortality by 2030 [1]. The only potentially curative treatment is surgical excision. However, by the time of diagnosis, due to advanced local progression or distant metastasis, pancreas cancer is frequently considered unresectable. Therefore, surgery is proposed as a viable option in only 10% to 20% of patients. Pancreatic resection is also the most complex abdominal operation, whose morbidity remains high with rates between 30 and 60% [2]. Postoperative complications such as surgical site infection, delayed gastric emptying (DGE), pancreatic fistula (POPF), post-pancreatectomy hemorrhage (PPH) and poor pain control are proved to be the main reasons for prolonged length of stay [3]. Even after successful pancreatic resection, the prognosis remains very poor. Early relapse and metastasis are not uncommon with a rate of recurrence between 65% to 95% of patients. Therefore, the 5-year survival rate of pancreatic cancer is approaching 20% after successful resection and chemotherapy. The median survival is between 18 and 29 months, ranking firmly last amongst all cancer sites outcomes for patients [2].

Recent reviews have emphasized the importance of the perioperative period on oncologic outcome after cancer surgery [4]. Indeed, the biological perturbations that accompany the surgical stress response and the pharmacological effects of anesthetic drugs, paradoxically, can promote disease recurrence or the progression of metastatic disease. This is possibly linked to the suppression of natural killer cell activity, which may be particularly important after pancreatectomy [5–7]. Many perioperative risk factors that can modulate surgery-induced immunosuppression have been suggested such as anesthetic technique, analgesic agents, blood transfusion, hypothermia, and pain. Adequate postoperative pain relief during the early postoperative period seems to carry the greatest clinical implications for oncologic outcomes after pancreatic resection [4, 5]. On the other hand, concerns have grown about unnecessary opioid use [8]. Nonetheless, opioids have been the mainstay of pain control after pancreas surgery. However, this approach is known to result in excess opioid consumption, potential narcotic dependence, respiratory depression, nausea, and vomiting, DGE and postoperative ileus. The latter two being known as the main drivers of length of stay after pancreatic surgery [9]. Therefore, every effort to minimize opioid use have been at the forefront with the implementation of opioid sparing strategies tailored to each institutional expertise as strongly recommended by the ERAS society [10]. Opioid-free anesthesia (OFA) is described but its feasibility and possible benefits when compared with opioid-based anesthesia (OBA) remain largely unexplored [11]. This study reviews the outcomes in a single-center cohort of patient who underwent pancreatic resections under OBA versus OFA.

## Methods

Data from patients who underwent pancreatectomy for tumors with curative intent at the Department of Abdominal Surgery and Transplantation, CUB Érasme, Free University of Brussels (ULB), from December 2019 to February 2021 were reviewed from a prospectively maintained database. Indeed, by law, data from all patients undergoing surgical resection for a suspected pancreatic or periampullary tumor must be communicated to a national advisory body funded by the federal government: the Belgian Healthcare Knowledge Center (KCE) [12]. The KCE aims to improve patient outcomes after pancreatic surgery by reducing practice variation and stimulating “best practices” [13]. Because of this mandatory declaration to the national cancer registry, a general agreement for using anonymous patient data was available and individual written consent was waived by the medical ethics Committee who authorized the utilization of OFA.

This single-institution retrospective cohort study was handled in accordance with the Declaration of Helsinki. This manuscript adheres to the applicable STROBE guideline.

### Anesthesia

All procedures were performed under general anesthesia with advanced vital sign monitoring, such as electrocardiogram, central venous pressures and invasive arterial blood connected to an advanced monitoring platform (Hemosphere, Edwards Lifesciences, Switzerland), SpO_2_, body temperature, and capnography.

Before induction, all patients received antibiotic prophylaxis, and intravenous (IV) loads of magnesium (30 to 40 mg/kg), dexamethasone (10 mg) and diclofenac (75 mg) according to the presence of a contraindication [14]. Patients from the opioid-based anesthesia group (OBA) also received a single bolus of intrathecal morphine (4 $\upmu$g/kg with a maximum of 300 $\upmu$g) [15].

The standardized IV induction included propofol (1.5 to 3 mg/kg), lidocaine (1.5 mg/kg bolus with a maximum of 100 mg) and rocuronium before tracheal intubation (0.6 to 1.2 mg/kg). In the OBA group a target-controlled infusion (TCI) of remifentanil (3–5 ng/ml) was used, while in the OFA group, patients received IV esketamine (0.25 mg/kg bolus) along with a continuous infusion of dexmetedomidine (0.5 μg/kg/h) started 10 min before induction.

In all patients, depth of anesthesia was individually adjusted to achieve and maintain a Bispectral Index between 40 and 60 (BIS, Covidien, France) with sevoflurane in an oxygen–air mixture to obtain a SpO_2_ $\ge$ 94%. Maintenance of anesthesia also included IV lidocaine 1.5 mg/kg/h, IV esketamine 0.125 mg/kg/h, and dexmetedomidine (0.4 to 0.7 μg/kg/h) in the OFA group, while remifentanil was used in the OBA group (2–5 ng/ml). A deep neuromuscular blockade was maintained during the whole procedure by iterative rocuronium bolus injection according to neuromuscular transmission monitor (ToFscan, IDMED, France). Dexmedetomidine, lidocaine or remifentanil were stopped at the end of surgery while esketamine was stopped 30 min earlier. All anesthetic drugs dosages were based on the adjusted body weight (ideal body weight + 0.4 (total body weight—ideal body weight) and then titrated to effect, except for neuromuscular blocking agent that was dosed according to the ideal body weight [16].

All patients were monitored in post-anesthesia care unit (PACU) for at least one night. They received a weight-adapted thrombosis prophylaxis with low molecular-weight heparin combined with compression stockings, and an IV pancreatic secretion inhibitor (somatostatin, 0.25 μg/h for 24 h). Patients were transferred to the ward if they were appropriately responsive or unchanged from preoperative status. Respiration had to be easy and unobstructed within 12 to 25 breaths/min. Heart rate and blood pressure had to be within acceptable range (60 to 100 beats/min and a MAP > 65 mmHg) with a SpO_2_ 94% or above on room air. Adequate urine output (0.5–1 mL/kg per hour) had to be maintained. NRS pain score had to be ≤ 4 before discharge based on a 0 to 10 pain scale with opioids administered no less than 15 min prior to discharge. Nausea and vomiting had to be under control.

Postoperative pain treatment was identical in both groups and performed by a combination of continuous wound infiltration [17] with ropivacaine 0.2% at 10 ml/h (Infusor; Baxter, Canada) for 48 h and patient-controlled analgesia with morphine (IV-PCA), followed by stepwise dose reduction and, finally, transition to nonopioid medication.

### Primary and secondary outcomes

The main objective of this study was to determine the benefit, if any, of the administration of OFA on postoperative pain control evaluated by numerical rating scale (NRS) and cumulated opioids consumption within 48 h after extubation. These two parameters were evaluated at several time points after extubation (1 h, 12, 24, and 48 h). Complications are also important indicators of immediate postoperative outcomes. Therefore, all adverse events within 90 days after surgery were recorded and evaluated by the surgical team according to the Clavien-Dindo classification (CDC), a widely used index for the classification of surgical complications and the comprehensive complication index (CCI) [18]. The CDC is a validated system which reports only the most severe complication while the CCI is instead designed to capture the overall burden of complications [19]. Length of stay and the 30-day mortality were also recorded.

Pharmacy data allowed for electronic abstraction of each opioid dose charted in the medication record. All routes of opioids (tramadol included) were summarized, including intermittent intravenous, oral, and IV-PCA totals. After collection of individual patient opioid doses, amounts were converted to IV morphine milligram equivalents (MME) using accepted conversion factors [20, 21]. For instance, 1 mg of IV MME was equivalent to 10 mg of parenteral tramadol, 30 mg of oral tramadol, or 0.7 mg of piritramide. Although conversion ratio also exists for intrathecal opioids (1:100), due to their site of action and time of administration, they were excluded from the total as they were not considered as systemic [22] nor as postoperative [23].

Since a recent study reported that OFA with dexmedetomidine might increase serious adverse events, mainly bradycardia with the possibility of asystole requiring cardiopulmonary resuscitation (CPR) [24], safety outcomes such as number of episodes of bradycardia requiring atropine administration, hypotension defined as mean arterial pressure lower than 65 mmHg and rescue medication (mainly continuous IV norepinephrine) were also recorded.

### Statistical Analysis

Dichotomous data were presented as proportions while continuous data were presented as median with interquartile range (IQR). After evaluation of the normality distribution with the Shapiro–Wilk test, difference between groups in continuous variables were assessed using either unpaired t-test or Mann–Whitney as appropriate. Differences in categorical variables were analyzed using Chi-square test. A multivariable regression model was used to determine the association of individual patient and surgical factors with LOS. Therefore, known significant factors for prolonged hospital stay such as age, BMI, gender, type of procedure, Wirsung dilatation, POPF and DGE [25] were also included. Regression coefficients ($\beta$) with 95% confidence intervals were computed. A *P* value of < 0.05 was considered significant for all statistical tests. Finally, Using the Gpower computer program [26], a post hoc power calculation was achieved to determine if the study was appropriately powered to detect outcomes. Statistical analysis was performed using GraphPad Prism version 9.1.0 for MacOS (GraphPad Software, San Diego, California USA).

## Results

A total of 77 consecutive patients were included in this analysis. Demographic and clinical characteristics of the two groups were similar at baseline. Females accounted for 39% of the total cohort. The median age was 67 [27–85] with 71.4% (*n* = 55) of patients older than 60 years. A high-risk comorbidity profile (ASA III–IV) was seen in 32.2% of the patients. Opioid-based anesthesia was administered in 42 patients (54.6%), while OFA was used in 35 (45.4%) patients. Fifty-four pancreaticoduodenectomy (PD – 70.1%), 21 distal pancreatectomies (DP – 27.3%), and 2 total pancreatectomies (2.6%) were performed by 3 specialized pancreatic surgeons. These resections were performed for malignant tumors in 73 patients (94.8%) and for benign tumors in 4 patients (5.2%).

### Primary Outcomes

**Fig. 1:**
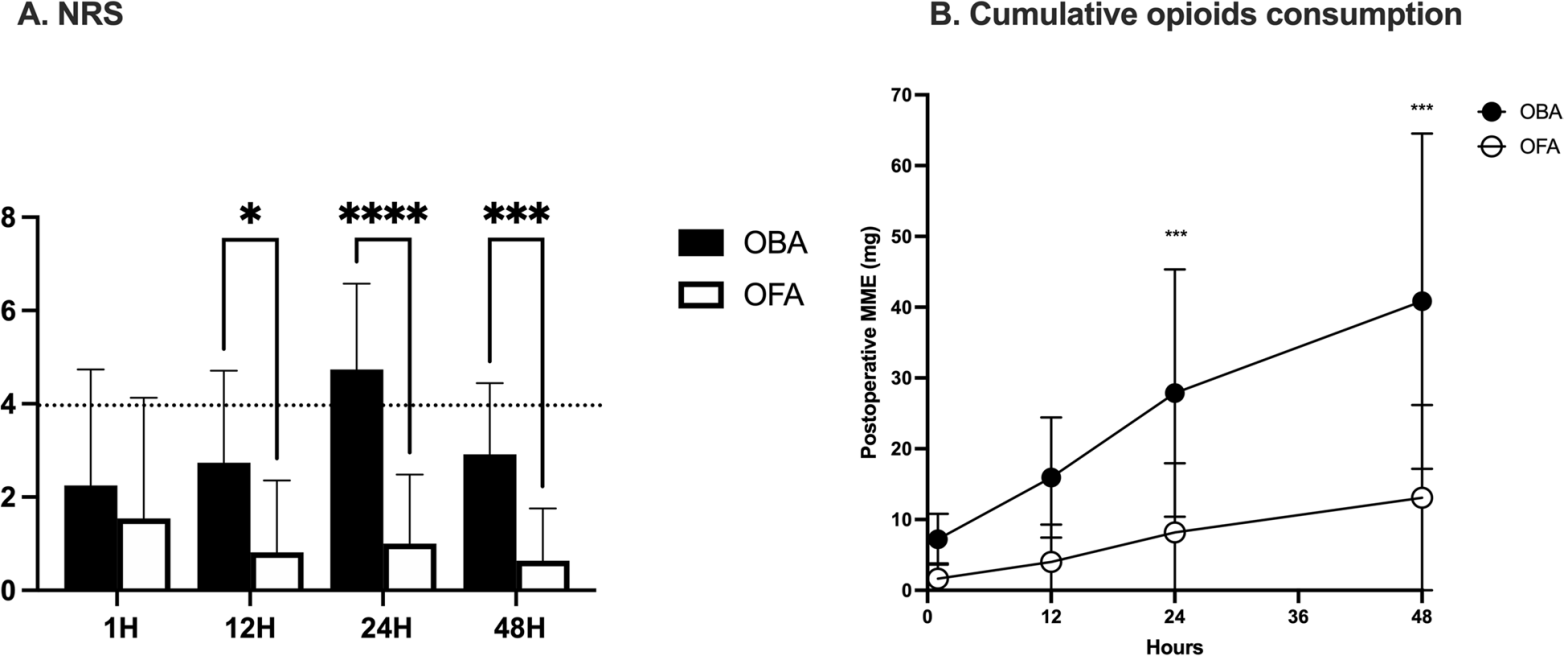
Longitudinal comparison of pain control after pancreatic surgery. **A** Numerical Rating Scale (NRS); **B** Cumulative Morphine Milligram Equivalent (MME). OFA: Opioid-free anesthesia; OBA: Opioid-based anesthesia (ns: *P* > 0.05; *: *P* < 0.05; **: *P* < 0.01; ***: *P* < 0.001; ****: *P* < 0.0001)

The cumulative postoperative morphine consumption and analgesia measures are given in Fig. 1. Compared to OBA, pain scores were significantly lower from 12 h and beyond (12 h: 3 [1–4] vs 0 [0–1]; median difference, 1.9; 95% CI, 0.1 to 3.7; *P* = 0.03; 24 h: 5 [4–6] vs 0 [0–2]; median difference, 3.7; 95% CI, 2 to 5.4; *P* = 0.005; 48 h: 3 [2–4] vs 0 [0–2]; median difference, 2.3; 95% CI, 0.9 to 3.6; *P* = 0.0003) while cumulative morphine consumption was only significant from 24 h and beyond (24 h: 24 [18.5–33] vs 4 [0–12]; median difference, 19.7; 95% CI, 6.8 to 32.6; *P* = 0.0007; 48 h: 36 [24–52] vs 10 [2–24]; median difference, 27.8; 95% CI, 10.5 to 59; *P* = 0.005). Three patients did not need any morphine administration after surgery in the OFA group and none in the OBA group.

### Secondary outcomes

Descriptive statistics comparing patients according to the anesthesia regimen and the overall cohort are shown in Table 1. The overall median operating time was 6 h [103–660]. Blood loss was at a median of 625 ml [50–3400] and 24.7% of the patients needed blood transfusion therapy. A median of zero units of packed red blood cells were used [0–12 units]. Typically, patients were transferred from the PACU to the ward within 24 h. The 30-day mortality and hospital readmission rate were set at 13 and 11.7% respectively.

**Table 1:** Patient, perioperative characteristics and outcomes

Characteristic	Total (*n* = 77)	OBA (*n* = 42)	OFA (*n* = 35)	*P*
**Demographic Features**				
**Age** (years)	67 [27–85]	68 [27–85]	66 [39–81]	0.66
**Female**	30 (39%)	20 (47.6%)	10 (28.6%)	0.23
**BMI** (kg/m^2^)	27 [18–39]	27 [18–39]	27 [20–36]	0.44
**Perioperative Factors**				
**Procedures**				
Pancreaticoduodenectomy	54 (70.1%)	31 (73.8%)	23 (65.7%)	0.96
Distal Pancreatectomy	21 (27.3%)	10 (23.8%)	11 (31.4%)	
Other	2 (2.6%)	1 (2.4%)	1 (2.9%)	
**Operative time** (minutes)	360 [103–660]	359 [166–660]	364 [103–544]	0.8
**Atropine**	7 (9.1%)	5 (11,9%)	2 (5,7%)	0.14
**Norepinephrine** ($\upmu$ g/kg/min)	0.043 [0–0.2]	0.049 [0–0.2]	0.037 [0–0.11]	0.73
**Blood loss** (ml)	625 [50–3400]	700 [100–2600]	490 [50–3400]	0.59
**Time to extubation** (minutes)	7.0 [1.5–47]	7.0 [1.5–42]	7.5 [2–47]	0.88
**Time to PACU discharge** (Hours)	18.2 [7.1–21.7]	19.3 [7.1–20.9]	18.3 [7.9–21.7]	0.94
**Length of stay** (days)	12 [6–46]	14 [7–46]	10 [6–16]	< 0.001***
**Index Complications**				
**Clavien-Dindo**				
None	7 (9.0%)	3 (7%)	4 (11.4%)	0.86
Grade I	31 (39.7%)	13 (30.2%)	18 (51.4%)	
Grade II	21 (26.9%)	15 (34.9%)	6 (17.1%)	
Grade III	11 (14.1%)	7 (16.3%)	4 (11.4%)	
Grade IV	5 (6.4%)	3 (7.0%)	2 (5.7%)	
Grade V	3 (3.9%)	2 (4.7%)	1 (2.9%)	
**CCI**	20.9 [0–100]	20.9 [0–100]	0 [0–100]	0.03*
**POPF**				
None	63 (81.8%)	33 (78.5%)	30 (85.7%)	0.76
Grade A	4 (5.2%)	2 (4.8%)	2 (5.7%)	
Grade B	9 (11.7%)	7 (16.7%)	2 (5.7%)	
Grade C	1 (1.3%)	0 (0%)	1 (2.9%)	
**DGE**				
None	66 (85.7%)	32 (76.2%)	34 (97.1%)	0.31
Grade A	2 (2.6%)	2 (4.8%)	0 (0%)	
Grade B	7 (9.1%)	6 (14.2%)	1 (2.9%)	
Grade C	2 (2.6%)	2 (4.8%)	0 (0%)	
**Readmission**	9 (11.7%)	8 (19%)	1 (2.9%)	0.09
**30-Day mortality**	10 (13%)	7 (16.7%)	3 (8.6%)	0.53

*OBA* Opioid-Based Anesthesia, *OFA* Opioid-Free Anesthesia, *CCI* Comprehensive Complication Index, *POPF* Post-Operative Pancreatic Fistula, *DGE* Delayed Gastric Emptying. (ns: *P* > 0.05; *: *P* < 0.05; **: *P* < 0.01; ***: *P* < 0.001; ****: *P* < 0.0001)

The only patient and surgical factors that were statistically different between the different anesthesia regimen was the length of stay and the CCI (*P* = 0.03). Compared to OBA, hospitalization was reduced by 4 days in case of OFA (14 [7–46] vs 10 [6–16] respectively, *P* < 0.001). Multivariable analysis identified OFA (*P* = 0.03), the absence of POPF (*P* = 0.0002) or a POPF requiring a change in the postoperative management ($\ge$ Grade B – *P* < 0.0001) [27], and DGE grade C (*P *< 0.0001) as significant independent factors for length of stay (Table 2).

**Table 2:** Multivariable analysis of factors associated with length of stay after pancreatic resection (OBA: 24.9 ± 25.5 days *vs* OFA: 14.1 ± 23.4 days, *P* = 0.03)

	${\varvec{\beta}}$	**95% CI**	***P***
**Age**	-0.06	-0.189 to 0.06	0.34
**Female**	0.65	-2.53 to 3.83	0.68
**BMI**	-0.008	-0.037 to 0.021	0.57
**Wirsung dilation**	0.24	-0.5 to 0.985	0.51
**Procedures**			
Pancreaticoduodenectomy	2.43	-1.46 to 6.31	0.21
Distal Pancreatectomy	-1.65	-5.71 to 2.4	0.41
**Anesthesia**			
Opioid-Free Anesthesia	-1.62	-5.01 to 1.78	0.03*
**POPF**			
None	-9.47	-14.2 to -4.73	0.0002***
$\ge$ Grade B	11.99	7.34 to 16.68	< 0.0001****
**DGE**			
Grade C	33.07	24.06 to 42.08	< 0.0001****

*POPF* Post-Operative Pancreatic Fistula, *DGE* Delayed Gastric Emptying. (ns: *P* > 0.05; *: *P* < 0.05; **: *P* < 0.01; ***: *P* < 0.001; ****: *P* < 0.0001)

### Safety Outcome

Bradycardia requiring atropine administration was more frequent in the OBA group than in the OFA group (11.9% vs 5.7%). However, this difference was not statistically significant (*P* = 0.14) and the maximal dose administered was 0.75 mg. As for atropine, need for hemodynamic support to achieve a MAP $\ge$ 65 mmHg was similar in all groups (OBA: 90.5%; OFA: 85.8%; *P* = 0.73, Chi-square test) with a median dose of 0.043 g$\upmu$/kg/min [0–0.2] also similar among groups (*P* = 0.73) (Table 1).

Median time to extubation were not different between groups (Table 1).

Finally, based on the mean, between-groups comparison effect size observed in the present study, the post hoc power analysis revealed that a total sample of 24 people using t-test and 48 people using chi-square would be needed to obtain statistical power at a 0.9 level with alpha at 0.05.

## Discussion

Surgery commonly causes postoperative pain that should be alleviated as soon and as effective as possible to reduce suffering, to promote the healing process and rehabilitation and to prevent complications. However, 80% of patients report their postoperative pain is not adequately treated, a metric unchanged for decades. Although debated [24], the utilization of opioid-free anesthesia (OFA) could be the answer. It is associated with a reduction of postoperative morphine consumption, and better pain control in selected patient and selected procedure [28]. Inspired by promising results, our anesthesia regimen was adapted to avoid intraoperative opioid use. This retrospective study shows that OFA is feasible even after major abdominal surgery with good results.

Consistent with other published reports, we found that introduction of an OFA protocol for pancreatic resection resulted in a 60% reduction in opioids requirement and NRS scores. Indeed, in the OBA group the NRS was almost systematically above the NRS cut-off for opioid administration (> 4) while a NRS pain scores ≤ 4 in the OFA group was the standard. However, there is large variation in how patients translate their pain to an NRS score. Therefore, without a pain assessment beyond the NRS, there is a risk of both undertreatment and overtreatment of the patient postoperative pain [29]. Since this was mitigated by the utilization of an IV-PCA, it allows us to assume that both these indicators give a fair picture of postoperative pain control. At least they should reduce the fear of more difficult postoperative pain control.

The time required until patients consistently report minimal postoperative pain is also key feature of pain control. It must be noted that NRS scores increased in the OBA group at 24 h but not in the OFA group. This might be explained by the anti-hyperalgesic techniques included in the OFA regimen, and/or by an opioid-induced hyperalgesia induced by OBA. These non-mutually exclusive hypotheses are supported by the fact that movement-related pain is substantially more intense than pain at rest and seems to be more closely associated with pain-related functional impairment [30]. The 24 h measurement correspond to the return to the ward. Therefore, pain intensity may have been influenced by activity such as sitting or standing. This movement-related pain, also called dynamic pain, has been suggested to be associated with opioid-induced hyperalgesia. Indeed, several laboratory and clinical studies have shown that hyperalgesia occurs after the administration of opioids (most of the time, after the termination of ultra-short-acting opioid remifentanil infusion). Although it have been reported that preincisional intrathecal morphine during spinal surgery allows for the control of increased postoperative analgesic requirements after intraoperative remifentanil [31], our results might suggest otherwise. Indeed, literature about intrathecal morphine before hepatic/pancreatic surgery reports equivocal findings. For some it may offer better postoperative pain control while others do not report any benefice over IV morphine [32]. When effective in abdominal surgeries, the literature suggests that there is no additional benefit after the first postoperative day [33]. It also suggests that its less efficient against dynamic pain compared to pain at rest. Both these features are consistent with our results. Finally, several case reports have demonstrated the possibility of hyperalgesia with intrathecal morphine. This might be related to the metabolization of morphine in morphine-3-glucoronide which is known to induce potent allodynia and hyperalgesia when injected intrathecally [34]. Independently of the putative mechanism of hyperalgesia, it could be prevented by the use of ketamine and alpha-2 agonists like dexmedetomidine [35].

Compared to OBA, with a discharge on median day 10, we report a shorter postoperative stay. This indicate that OFA might be more than a feasible option but also a viable one. In our cohort, independent variables associated with extended length of stay included, as expected, the presence of index complications mainly POPF $\ge$ grade B and DGE grade C [3]. This study confirms that index complications are important indicators of immediate postoperative outcomes. Indeed, the CCI known for its significantly stronger correlations with LOS and cost of complications [18], was the lowest in the OFA group and statistically different from the OBA group. This is of importance since the CCI at postoperative day 7 strongly predicts high 90-day morbidity (odds ratio 3.96 per 10 CCI points, *P* < 0.001) [36]. Although not significant, the fewest index complications, especially POPF and DGE, were seen in the OFA group. The reduction in DGE make sense, as drug-induced gastric emptying delay is commonly reported in patients receiving opioids for postoperative pain management. Moreover, numerous studies have shown the opioid-sparing effect of a multimodal approach combining regional analgesia, non-opioid analgesics, lidocaine infusions, and ketamine, as in the OFA group, resulted in an accelerated gastrointestinal recovery and improved outcomes [37]. A recent study identified an inverse correlation between length of stay and readmission, prolonged length of hospital stay being protective for certain post-discharge complications requiring readmission, mostly surgical site infection [38]. In our setting, reduced length of stay with OFA did not lead to the unintended consequence of increasing readmission rates, neither in an increased 30-day mortality or unexpected post-discharge complication. It is however too early to identify long-term benefit such as improved disease-free or overall survival due to the limited follow-up (upmost 13 months) [4, 39].

Recently, OFA with dexmetedomidine was associated with more adverse events such as bradycardia, asystole, hypoxemia delayed extubation, and prolonged PACU stay, despite a lower overall opioid consumption and less postoperative nausea and vomiting [24]. Most of these adverse events were not present in our setting. This might be explained by several factors. First, definition of bradycardia by Beloeil et al.was rather liberal, while we only treated bradycardia, provide that the MAP was maintained, if heart rate was < 40 bpm, supported by data showing that a slower heart rate (< 55 bpm) has been associated with reduced risk for myocardial injuries in non-cardiac surgeries as well as mortality [40]. Second, according to the internal guidelines and published recommendations, we provided preemptive oxygen therapy [24]. Finally, the dosage of dexmedetomidine was much lower and began with a loading dose. Dose differences may be associated with different effects, sometimes even more important than the medication choice. For instance, regarding the extubation delay and postoperative sedation, some studies reported a reduced delay [41], while other reported the opposite [42]. In this study, we report no difference between groups. This might be the consequence of a resulting mean dosage of the continuous infusion of dexmetedomidine at 0.5 ± 0.2 μg/kg/h, where the POFA study reported a median and a mean dose of respectively 0.9 and 1.2 μg/kg/h, but, importantly, without including any loading dose. Therefore, we could hypothesize that the absence of increased sedation, delayed extubation and the absence of severe bradycardia observed in our study are a consequence of this adequate loading dose conjointly with a low maintenance.

According to some authors, the intentional adoption of perioperative lidocaine infusion over an extended time frame could increase the likelihood for local anesthetic systemic toxicity leading to a virtual certainty for clinically significant, even fatal events [43]. Although the present literature does not confirm this possibility, we must keep in mind that toxicity from perioperative lidocaine infusion is exceedingly rare [44]. Therefore, this author advocates for point of care serial lidocaine blood concentration determinations on all patients [43]. This has already been done in colorectal [45] and bariatric surgery [46]. Although there was a wide range of plasma concentrations up to 10 g/ml, when the infusion protocol for lidocaine is based on an adapted body weight rather than actual body weight (as in our setting), plasma concentrations were inside the usual accepted safe range from 1.5 to 5 µg/ml in both studies. Consistently with those results, no adverse events or reports of symptoms of local anesthetic toxicity were recorded in the medical files.

Limitations of these work are linked to the retrospective, single-institution cohort design with a relatively limited number of patients. Our sample size was one of convenience, chosen from the time of first OFA implementation. However, to limit selection bias, we analyzed consecutive patients. Also, based on the post hoc power calculation, it is unlikely that our findings can be attributed to the limited sample size. However, even if an inclusion bias is controlled, the non-blind assessment by nurses may have influenced results and other confounders cannot be formally excluded, including the anesthesiologist in charge, the day of the week, among others. Determining the incidence of any opioid related side effects in PACU and/or on the ward would also have added value to this study. However, the retrospective nature of the analysis prevented us to obtain these information’s. More in case of suspected side effects based on some prescriptions such as antiemetic, the causal relationship could not be affirmed. Despite these limitations, we believe that our findings add information about feasibility, safety, and viability of OFA in major abdominal surgery. It shows that, in experienced hands, the technique is deemed appropriate, and potentially better than the others, even if demonstrating a direct effect of OFA will require a randomized-controlled trial.

## Conclusion

The implementation of an OFA protocol was associated with a better pain control as measured by pain rating at 24 and 48 h and cumulative opioid dose, a reduced comprehensive complication index compared to OBA, and a 4-day reduction in length of stay after pancreatic resection without an increase in morbidity or readmissions. No adverse hemodynamic effect was suspected in the OFA group.

## Data Availability

The datasets used and analyzed during the current study are available from the corresponding author on reasonable request.

## Abbreviations

OFA: Opioid-free anesthesia
OBA: Opioid-based anesthesia
DGE: Delayed gastric emptying
POPF: Postoperative pancreatic fistula
PPH: Post-pancreatectomy hemorrhage
KCE: Belgian healthcare knowledge center
PACU: Post-anesthesia care unit
NRS: Numerical rating scale
CCI: Comprehensive complication index
MME: Morphine milligram equivalents
MAP: Mean arterial pressure
