# Correction to: Opioid-free versus opioid-based anesthesia in pancreatic surgery

**DOI:** 10.1186/s12871-022-01572-1

**Published:** 2022-01-22

**Authors:** Stéphane Hublet, Marianne Galland, Julie Navez, Patrizia Loi, Jean Closset, Patrice Forget, Pierre Lafère

**Affiliations:** 1 Department of Anesthesiology, Université Libre de Bruxelles, CUB Érasme, Brussels, Belgium; 2 Department of Abdominal Surgery and Transplantation, Université Libre de Bruxelles, CUB Érasme, Brussels, Belgium; 3 Clinical Chair in Anaesthesia, University of Aberdeen, Aberdeen, UK

**Correction to: BMC Anesthesiol 22, 9 (2022)**

**https://doi.org/10.1186/s12871-021-01551-y**

Following publication of the original article [1], the authors reported an error in the presentation of the names wherein the given and last names has been swapped. The correct presentations are as follows:

Given name: Stéphane, Last name: Hublet

Given name: Marianne, Last name: Galland

Given name: Julie, Last name: Navez

Given name: Patrizia, Last name: Loi

Given name: Jean, Last name: Closset

Given name: Patrice, Last name: Forget

Given name: Pierre, Last name: Lafère

The original article [1] has been updated.
